# Health-Related Quality of Life and Anxiety Levels in Pregnant Women with and Without Associated Pathologies

**DOI:** 10.3390/jcm14196815

**Published:** 2025-09-26

**Authors:** Brenda-Cristiana Bernad, Mirela-Cleopatra Tomescu, Dana Emilia Velimirovici, Minodora Andor, Diana Lungeanu, Virgil Enătescu, Andreea Luciana Rață, Sergiu-Florin Arnăutu, Andreea Sălcudean, Oana Neda-Stepan, Lavinia Hogea

**Affiliations:** 1 Doctoral School, “Victor Babes” University of Medicine and Pharmacy from Timișoara, 300041 Timișoara, Romania; bernad.brenda@umft.ro; 2 Department of Neuroscience, “Victor Babes” University of Medicine and Pharmacy from Timișoara, 300041 Timișoara, Romania; enatescu.virgil@umft.ro (V.E.); oana.neda-stepan@umft.ro (O.N.-S.); hogea.lavinia@umft.ro (L.H.); 3 Multidisciplinary Heart Research Centre, “Victor Babes” University of Medicine and Pharmacy from Timișoara, 300041 Timișoara, Romania; tomescu.mirela@umft.ro (M.-C.T.); andor.minodora@umft.ro (M.A.); 4 Centre for Neuropsychology and Behavioural Medicine, “Victor Babes” University of Medicine and Pharmacy from Timișoara, 300041 Timișoara, Romania; 5 Department of Internal Medicine, “Victor Babes” University of Medicine and Pharmacy from Timișoara, 300041 Timișoara, Romania; 6 Department of Cardiology, “Victor Babes” University of Medicine and Pharmacy from Timișoara, 300041 Timișoara, Romania; dana.velimirovici@umft.ro; 7 Institute of Cardiovascular Diseases Timisoara, 300310 Timișoara, Romania; 8 Cardiology Clinics, Timisoara Clinical Municipal Emergency Hospital, 300040 Timișoara, Romania; 9 Center for Modeling Biological Systems and Data Analysis, Department of Functional Sciences, “Victor Babes” University of Medicine and Pharmacy from Timișoara, 300041 Timișoara, Romania; dlungeanu@umft.ro; 10 Clinic of Psychiatry, “Pius Brînzeu” County Clinical Emergency Hospital, 300723 Timișoara, Romania; 11 Department of Surgical Emergencies, “Victor Babes” University of Medicine and Pharmacy from Timișoara, 300041 Timișoara, Romania; andreea.rata@umft.ro; 12 Department of Vascular Surgery, “Pius Brînzeu” County Clinical Emergency Hospital, 300723 Timișoara, Romania; 13 Centre for Cognitive Research in Neuropsychiatric Pathology, “Victor Babes” University of Medicine and Pharmacy from Timișoara, E. Murgu Sq., no.2, 300041 Timișoara, Romania; 14 Department of Complementary Functional Sciences, “George Emil Palade” University of Medicine, Pharmacy, Sciences and Technology, 540142 Târgu Mureș, Romania; andreaa.salcudean@umfst.ro

**Keywords:** pregnancy, pregnancy-associated pathologies, anxiety, health, quality of life

## Abstract

**Background:** Since quality of life encompasses social, psychological, and physical well-being, it is a crucial component of overall health and well-being. The quality of life has a significant impact on both the mother and the unborn child throughout the perinatal period. Both parties suffer when a threat, such as an illness, materialises because it lowers the quality of life. Using the SCL-90-R and SF-36, the current study aims to investigate variations in anxiety levels and health-related quality of life (HRQoL) between pregnant women with and without relevant medical conditions. **Methods**: We carried out a cross-sectional study between April 2023 and December 2024. Eligibility criteria were: (a) pregnant women; (b) at least 18 years old; (c) of Romanian nationality residing in Romania; and (d) who signed informed consent and agreed to participate. A Personal Information Form (PIF), the SF-36 Health Survey, and the SCL-90-R questionnaire were used to collect data. Statistical analyses were performed with SPSS v26, using non-parametric tests (Mann–Whitney U, Spearman correlations). **Results:** Ninety-five of the 212 patients in the study reported having related medical conditions. There were no statistically significant differences between the groups in the physical or mental components of the SF-36. Nonetheless, the pathological group’s anxiety scores were noticeably higher. Particularly in the pathological group, Spearman correlation revealed an inverse relationship between anxiety and SF-36 physical component scores. **Conclusions:** The findings highlight the importance of integrating psychological screening into prenatal care, particularly for women with medical comorbidities. Early identification and management of elevated anxiety may help preserve maternal HRQoL and contribute to better perinatal outcomes.

## 1. Introduction

Health-related quality of life (HRQoL) is a concept that narrows the broader idea of quality of life, focusing on health-related factors. It encompasses various aspects of well-being and functioning, including psychological, social, role functioning, and physical domains. HRQoL reflects both subjective and objective views on how health status influences daily life. Generally, HRQoL aims to capture the effects of diseases and treatments on disability, functioning, and perceived health, thereby connecting medical conditions with overall quality of life [1,2].

HRQoL during pregnancy has been increasingly investigated, as the perinatal period represents a vulnerable stage in which both physical changes and psychological distress can significantly affect maternal well-being [3]. However, most studies have focused on general populations of pregnant women, with fewer examining the role of comorbidities or trimester-specific differences.

Pregnancy is a complex period, characterised by significant physiological, hormonal, psychological, and social changes that can impact the quality of life for pregnant women. In the context of pregnancy, HRQoL is influenced not only by physical factors but also by psychological aspects such as anxiety, stress and perception of the maternal role. Maternal stress and anxiety are essential psychological factors that significantly influence well-being and HRQoL during pregnancy and postpartum. These psychological dimensions are frequently interconnected with physical health, resulting in a complex, dynamic, and bidirectional impact on overall quality of life. Emotional and informational support provided by family, partner, friends, and healthcare professionals plays a fundamental role in reducing maternal stress and anxiety, thus contributing to improving HRQoL [4,5].

Anxiety is one of the most common emotional reactions encountered in the prenatal period, affecting between 10% and 20% of pregnant women [6]. High levels of anxiety are associated with several adverse outcomes for both the mother and the fetus, including obstetric complications, increased risk of postnatal depression, and decreased perceived quality of life [7,8]. Therefore, simultaneous measurement of HRQoL and anxiety levels provides a comprehensive perspective on the well-being of pregnant women.

In addition, the presence of comorbidities, such as gestational diabetes, hypertension, or preeclampsia, can exacerbate the negative impact of pregnancy on quality of life. These medical conditions require additional treatments, constant monitoring, and increased concern for the health of the fetus, which can exacerbate anxiety levels and reduce HRQoL [9,10].

Thus, comparing pregnant women with and without associated pathologies is particularly relevant to understanding differences in HRQoL and psychological state during pregnancy. The present study aims to assess and compare anxiety levels and HRQoL of pregnant women with and without associated medical conditions, to identify possible needs for additional support, both medical and psychological, in prenatal care. In line with this purpose, the study aimed to find answers to the following questions: Are there differences in the levels of anxiety between pregnant women with and without associated pathology? Are there differences in the levels of HRQoL between pregnant women with and without associated pathology?

## 2. Materials and Methods

### 2.1. Study Design and Setting

We conducted a cross-sectional study. We selected the study group from patients admitted to the Ist Obstetrics and Gynaecology Clinic (OGC) of the “Pius Brânzeu” County Emergency Clinical Hospital (PBCCEH), Timișoara, Romania, between April 2023 and December 2024. Only patients who met the eligibility criteria and who agreed to participate after being informed about the study procedure and signed a written informed consent for participation were included in the study. Subsequently, a member of the study medical staff instructed eligible participants about the study and what each questionnaire assessed. After signing the consent form, participants were given the questionnaires to complete. Completing the questionnaires took approximately forty minutes.

### 2.2. Participants

**Figure 1:**
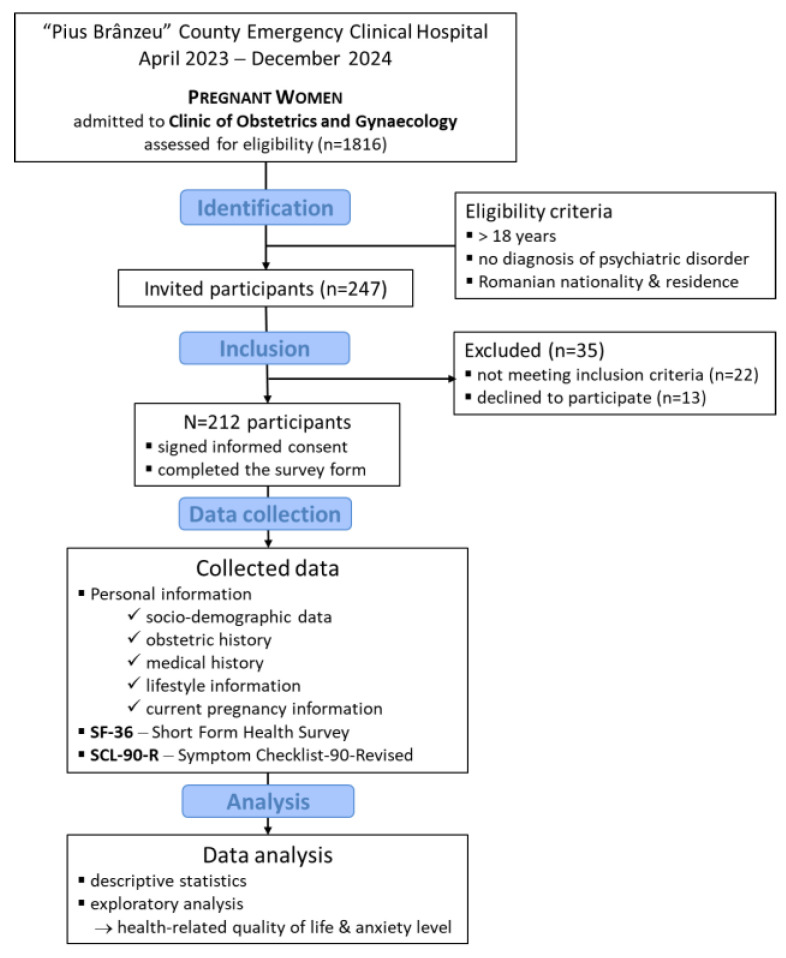
STROBE Flow Diagram of participant selection.

In the study, the patients who met the following eligibility criteria were included: (a) pregnant women; (b) at least eighteen years old; (c) of Romanian nationality residing in Romania; (d) who signed informed consent and agreed to participate in the study. Patients diagnosed with any psychiatric disorder were excluded from the study; this criterion was determined based on information from the participants’ medical records. Patients who did not meet the eligibility requirements and those who chose not to complete or only partially complete the questionnaires were excluded. Finally, the study included 212 pregnant women. The STROBE flow diagram illustrates the selection process of participants (Figure 1).

The 212 participants in the study were divided into two groups: those without associated pathologies and those with associated pathologies.

The pregnant women in the group with associated pathologies presented: bronchial asthma, chronic bronchitis, chronic hypertension or gestational hypertension, type 2 diabetes or gestational diabetes, hyperthyroidism or hypothyroidism, tachycardia, anaemia, peripheral varicose veins or gastroduodenal ulcer.

The indications for hospitalization of the patients included in the study in the second trimester admissions were linked to miscarriage risk, cervical insufficiency, bleeding of various causes (placenta praevia, early retroplacental hematoma), gestational hypertension, chronic hypertension, metabolic imbalances (uncontrolled diabetes, hyperthyroidism, ketoacidosis), severe hyperemesis gravidarum, diagnostic procedures, while, in the third trimester, admissions were linked to delivery planning, onset of spontaneous labor at term, premature rupture of membranes at term, hypertensive disorders, and severe anemia.

### 2.3. Data Collection

In the evaluation of this cross-sectional study, we utilised instruments from the Department of Neurosciences, the Centre for Neuropsychology and Behavioural Medicine, the Centre for Social Diagnosis, the Psychiatry Clinic, and the COG in Timisoara. Three questionnaires were applied to collect data for the study, which are described below.

(1) Personal Information Form (PIF)

The PIF was created by researchers, by reviewing the related literature [11,12,13,14,15], and included the following sections: (1) socio-demographic (age, education, place of residence, marital status, and employment status); (2) obstetric history (gravidity, parity, miscarriages, gestational age); (3) medical history—chronic diseases (hypertension, diabetes, thyroid, and so on); (4) lifestyle—smoking, alcohol; (5) current pregnancy details—complications—e.g., gestational diabetes, gestational hypertension.

(2) Short-Form Health Survey (SF-36)

SF-36 is a standardised self-report questionnaire on perceived health, developed initially as part of the Medical Outcomes Study [16]. The instrument consists of 36 items that assess eight dimensions of health-related quality of life: physical functioning, limitations due to physical health, bodily pain, general health, vitality, social functioning, limitations due to emotional problems, and mental health. Based on these dimensions, two composite scores can be calculated: the Physical Component Summary (PCS) and the Mental Component Summary (MCS), which provide a global picture of the individual’s physical and psychological health. Each dimension is converted into a score on a scale from 0 to 100, where higher scores indicate better perceived health.

In this study, the SF-36 questionnaire was used to assess the subjective perception of health status, utilising both the raw scores of each dimension and the two composite scores for global interpretation.

(3) SCL-90-R

The Symptom Checklist-90-Revised (SCL-90-R) is a self-report instrument developed by Derogatis (1994) [17] and widely used to measure psychopathological symptoms in a clinical or research setting. SCL-90-R contains 90 items, rated on a 5-point Likert scale, from 0 (“not at all”) to 4 (“extremely”), reflecting the degree of psychological distress experienced in the past seven days. The SCL-90-R contains nine clinical dimensions: somatisation, obsessions–compulsions, interpersonal sensitivity, depression, anxiety, hostility, phobic anxiety, paranoid anxiety, and bizarre ideation. In addition, the instrument generates three global indices of psychological distress: the Global Severity Index (GSI), the Positive Symptom Total (PST), and the Positive Symptom Distress Index (PSDI).

In the present study, the SCL-90-R was used to assess the psychological symptomatology of the participants, with scores being interpreted according to international norms. In the present study, we used only the anxiety subscale, which presented a Cronbach’s α of 0.83.

### 2.4. Ethical Approval

The study has been approved by the Scientific Research Ethics Commission of “Victor Babes” University of Medicine and Pharmacy, Timisoara (45/2 October 2023). We obtained the written consent from participants after they were informed of the study’s goal, prior to completing the questionnaires.

### 2.5. Data Analysis

The Statistical Package for Social Sciences (SPSS) version 26 (IBM Corp, Chicago, IL, USA) was used to analyse the data. Descriptive and exploratory statistical analyses were part of the data analysis. Missing data were handled using listwise deletion, with participants excluded from specific analyses if relevant values were not available. To determine the best type of analysis for testing hypotheses, we conducted preliminary descriptive analyses and assessed the normality of the distributions using the Shapiro–Wilk test. As the data did not meet normality assumptions, non-parametric methods, such as Spearman correlation and Mann–Whitney U analysis, were applied. Effect size (r) was calculated as Z/√N. A *p*-value < 0.05 was considered statistically significant.

## 3. Results

The patients were included in two groups: pregnant women without associated pathology (*n* = 117) and those with associated pathologies (*n* = 95). The analysis of socio-demographic, clinical, and lifestyle characteristics did not reveal statistically significant differences between pregnant women with and without associated pathologies. We observed that both groups were comparable in terms of age, educational level, marital status, residential environment, socio-economic status, and gestational age. Similarly, lifestyle factors such as smoking and alcohol consumption did not show significant variations, although a slightly lower smoking rate and higher alcohol consumption were observed among pregnant women with associated pathologies. Although not statistically significant, these observations indicate a tendency toward lifestyle differences that may merit further exploration in larger studies. These findings indicate a balanced distribution of variables, allowing a valid comparative analysis of the results between the two groups. Table 1 and Table 2 present the socio-demographic data of the entire sample and the two groups. Furthermore, Table 2 presents comparative analyses at the group level, taking into account socio-demographic data.

Participants with and without concomitant diseases (healthy group) were compared in terms of anxiety (measured using the SCL-90 anxiety subscale) and health-related quality of life (measured using the SF-36 mental component and physical component) using a Mann–Whitney U test (Table 3 and Table 4). While the small sample size limits the detection of robust statistical differences, the following results reveal meaningful patterns that may indicate clinically relevant trends.

For the mental component of the SF-36, there was no significant difference between the groups (U = 5474.50, *p* = 0.852), indicating that the perceived levels of this dimension are similar. Similarly, there was no significant difference in the physical component of SF-36 scores between the groups (U = 5163.00, *p* = 0.374). However, the group with associated pathologies consistently showed slightly higher mean ranks for physical health, suggesting a potential trend toward lower perceived physical well-being among healthy participants, despite the lack of statistical significance.

A significant difference was found for anxiety measured by the SCL-90-R (U = 4671.00, *p* = 0.044).

The mean ranks were higher for the group with associated pathologies (mean Rank = 115.83) compared to the group without associated pathologies (Mean Rank = 98.92), suggesting a lower level of anxiety in healthy participants. This pattern points to a consistent trend toward higher anxiety levels among women with associated conditions, even though the effect size remained small.

The effect size, calculated with the formula r = Z/√N, was r = 0.14, 95% CI [−0.27, −0.00], indicating a small magnitude effect according to Cohen (1988) [18].

An additional analysis is presented in Table 5. The associations between anxiety levels and mental and physical health were investigated using a Spearman correlation analysis. No significant association was found between mental health and anxiety (*r_s_* = 0.083, *p* = 0.374). However, physical health showed significant negative associations with anxiety as measured by SCL-90 (*r_s_* = −0.164, *p* = 0.002).

We conducted a Spearman correlation analysis within the pathological group (*n* = 95) to explore the relationships between physical and mental health of SF-36 and anxiety levels, as shown in Table 6. Results indicated a significant negative correlation between physical health and anxiety as measured by the SCL-90 (*r_s_* = −0.267, *p* = 0.009), suggesting that lower perceived physical health is associated with higher anxiety levels. Additionally, a significant negative correlation was found between mental and physical health components (*r_s_* = −0.258, *p* = 0.012).

Taken together, although many results did not reach conventional levels of statistical significance, the consistent direction of effects suggests clinically meaningful tendencies that warrant further investigation in larger cohorts.

## 4. Discussions

The present study investigated the relationships between perceived physical and mental health, measured by the SF-36, and anxiety levels assessed with SCL-90, in two groups: pregnant women without associated pathology (*n* = 117) and those with associated pathologies (*n* = 95). Some of our results support previous findings, while others diverge from them.

Initial analyses using the Mann–Whitney U test revealed no significant differences between the two groups in terms of the scores for the mental and physical components of the SF-36. Previous studies have shown that pregnant women with pathologies such as preeclampsia, metabolic syndrome, gestational hypertension, or urinary incontinence have significantly lower quality of life, both physically and mentally, compared to healthy women [19,20]. In particular, in the case of hypertensive disorders occurring during pregnancy, the data suggest a high psychological impact. Women with gestational hypertension presented significantly higher anxiety scores on the SCL-90 than those without such complications [21]. These trends confirm the robustness of the relationship between the existence of a pregnancy-related pathology and the increased level of psychological distress.

Significant differences were observed in the analysis of anxiety levels, with the pathological group presenting significantly higher scores on the SCL-90 anxiety scale. These results, which are consistent with the literature, provide a reliable understanding of the issue. They show that women with pregnancy-induced medical conditions experience significantly higher levels of anxiety compared to those with uncomplicated pregnancies [22,23,24], further validating this by comparing pregnant women diagnosed with hypertensive disorders to those without complications, finding significantly higher anxiety levels in the former. Pregnant women with medical conditions or high-risk pregnancies generally report higher levels of anxiety symptoms, and our research underscores the role of associated pathology as a risk factor for prenatal anxiety [25].

The SCL-90 instrument has proven valuable in detecting psychological distress, particularly among women experiencing pregnancy-related medical conditions [26]. It has been successfully used in numerous studies comparing healthy pregnant women with those with pathologies such as metabolic syndrome, gestational hypertension, or other obstetric complications [21,27].

Correlation analysis using Spearman’s rho on the entire study group (*N* = 212) showed no significant associations between the mental components of the SF-36 and anxiety measures. In contrast, the physical health component was negatively correlated with anxiety levels, suggesting that lower perceived physical health is associated with higher anxiety. This finding is consistent with the biopsychosocial model of health, which postulates a dynamic interaction between physiological conditions and psychological responses [28]. Pregnant women experiencing physical limitations or discomfort may internalise these changes as stressful, particularly if the complications pose potential risks to fetal outcomes. These associations have been corroborated in studies evaluating women with preeclampsia [29].

Spearman correlation was used to explore the dynamics within the comorbidity group. Within this group (*n* = 95), the physical health component was strongly negatively correlated with anxiety as measured by the SCL90. The mental health component was negatively and significantly correlated with the physical health component but showed no significant association with anxiety scores. This counterintuitive result shows that, in this group, higher mental well-being was associated with lower physical functioning. However, mental components of the SF-36 did not demonstrate any significant association with any of the anxiety scales, indicating that perceived mental health in this group may be relatively independent of acute anxiety symptoms. This paradoxical result may reflect psychological adaptation processes. In late pregnancy, physical discomforts are often normalized as a natural experience, while social support and anticipation of childbirth can enhance perceived mental well-being. This interpretation is consistent with the concept of “response shift,” whereby individuals adapt their internal standards when faced with physical limitations [30]. Similar findings have been reported in previous studies, where physical HRQoL declined but mental HRQoL remained stable or improved during the third trimester [3,31].

The absence of HRQoL differences between groups despite higher anxiety levels may be explained by the limited sensitivity of the SF-36 in late pregnancy [3,32], the strong overlap between anxiety and depressive symptoms during pregnancy [33], and the tendency of women to perceive anxiety as a common aspect of pregnancy, particularly when supported by family and healthcare providers [34].

This study has several strengths, including the use of validated instruments (SF-36 and SCL-90-R) and a balanced distribution between groups. Nonetheless, some limitations should be considered. First, the cross-sectional design prevents causal inferences. Second, all measures were self-reported, which may introduce bias and be influenced by cultural perceptions. The SF-36 may also lack sensitivity to subtle changes in HRQoL during late pregnancy or hospitalization. Most participants were in the third trimester, which limited the power of trimester-specific comparisons and subgroup analyses (e.g., hypertension vs. diabetes). Potential confounders, such as treatments, medication, and social support, were not controlled for. Additionally, the sample was restricted to one Romanian center, which may limit its generalizability to other settings; however, the “Pius Brânzeu” County Emergency Clinical Hospital is a tertiary care hospital affiliated with the “Victor Babes” University of Medicine and Pharmacy, and the Obstetrics and Gynaecology Clinic admits cases from all over Western Romania, with no bias or constraints regarding the diagnoses or severity of the medical condition. Finally, no a priori sample size calculation or power analysis was performed, which may limit the interpretation of non-significant HRQoL results.

Moreover, the relatively small sample size inevitably reduces statistical power and limits the ability to detect robust differences between groups. Nevertheless, by reporting both the numerical outcomes and the observed directions of change, we aimed to highlight emerging trends and potential associations that may hold clinical relevance. We therefore interpret our findings as exploratory and hypothesis-generating, emphasizing the need for confirmation in larger, adequately powered studies.

## 5. Implications

The present study offers several important contributions in the context of perinatal mental health care.

First, it provides empirical evidence that pregnant women experiencing pathological pregnancies report significantly increased levels of anxiety. The use of a validated symptom-specific instrument is necessary for routine psychological assessments in obstetric protocols.

Second, the study identifies a dissociation between health-related quality of life, assessed by the SF-36, and psychological distress, measured by the SCL-90-R. Although SF-36 mental and physical component scores did not differ significantly between groups, anxiety levels were significantly higher among those with medical complications. The results suggest that general quality of life assessments may be insufficient to detect acute psychological symptoms in high-risk pregnancies.

Third, the results indicate that perception of physical health is inversely associated with anxiety. Pregnant women who perceive themselves to be in poorer physical health are more likely to experience psychological distress. This highlights the importance of monitoring subjective perceptions of health in conjunction with objective clinical assessments.

Together, these contributions highlight the need for integrated, multidisciplinary models of prenatal care that prioritise both physical and mental health to improve maternal and fetal outcomes. Timely identification of anxiety symptoms can enable prompt psychosocial interventions, which have been shown to enhance both maternal well-being and birth outcomes [35].

## 6. Conclusions

Pregnant women with associated pathologies present significantly higher levels of anxiety compared to healthy women, although no significant differences in HRQoL were found. Clinically, our results highlight the importance of incorporating validated psychological screening instruments, such as the SCL-90-R or other perinatal-specific measures (e.g., EPDS, STAI), into routine prenatal care, especially for patients with identified medical risks. Early identification of anxiety symptoms may facilitate timely psychosocial interventions, which are known to improve both maternal and birth-related outcomes.

Future studies should investigate longitudinal trajectories of anxiety and HRQoL across pregnancy and postpartum and complement quantitative results with qualitative insights into perceived support, health literacy, and cultural attitudes.

## Figures and Tables

**Table 1:** Socio-demographic characteristics of the participants.

Baseline Characteristic		Full Sample	
		*N*	%
Age group			
	≤30 years	105	49.5
	>30	107	50.5
Residence type			
	Urban	110	51.9%
	Rural	102	48.1%
Educational level			
	Primary school	4	1.9
	Middle school	9	4.2
	Vocational school	21	9.9
	High school	86	40.6
	Graduate school	64	30.2
	Postgraduate degree	28	13.2
Working conditions			
	Low-risk	158	74.5
	Medium-risk	42	19.8
	High-risk	12	5.7
Socio-economic conditions			
	Very good	44	20.8
	Good	121	57.0
	Satisfying	47	22.2
	Poor	0	0
Trimester of pregnancy			
	II	28	13.2
	III	184	86.8
Associated pathologies			
	No	117	55.2
	Yes	95	44.8

**Table 2:** Socio-demographic and clinical characteristics of the pregnant patients from the two groups.

Patient Characteristics	Without Diseases	With Associated Diseases	*p* Value ^(b), (c)^
**Variable** ^(a), (b), (c)^	*N* = 117	*N* = 95	
**Socio-demographic characteristics**			
**Maternal age [years]** ^(a)^	30.08 ± 5.86	30.99 ± 6.67	0.386 ^(c)^
**Education level** ^(b)^			
Primary school	3 (2.6%)	1 (1.1%)	0.944
Middle school	5 (4.3%)	4 (4.2%)	
Vocational school	10 (8.5%)	11 (11.6%)	
High school	47 (40.2%)	39 (41.1%)	
Graduate school	36 (30.8%)	28 (29.5%)	
Postgraduate degree	16 (13.7%)	12 (12.6%)	
**Rural area** ^(b)^	57 (48.7%)	45 (47.4%)	0.867
**Marital status** ^(b)^			
–Married	95 (81.2%)	80 (84.2%)	0.711
–Single	16 (13.7%)	12 (12.6%)	
–Divorced/Widowed	6 (5.1%)	3 (3.2%)	
**Working conditions** ^(b)^			
Low-risk	91 (77.8%)	67 (70.5%)	0.166
Medium-risk	18 (15.4%)	24 (25.3%)	
High-risk	8 (6.8%)	4 (4.2%)	
**Socio-economic conditions** ^(b)^			
Very good	27 (23.1%)	17 (17.9%)	0.155
Good	70 (59.8%)	51 (53.7%)	
Satisfying	20 (17.1%)	27 (28.4%)	
**Gestational age** ^(b)^			
2nd Trimester	62 (53.0%)	50 (52.6%)	0.978
3rd Trimester	55 (47.0%)	45 (47.4%)	
**Lifestyle**			
Smoking during pregnancy ^(b)^	31 (26.5%)	16 (16.8%)	0.129
Alcohol consumption ^(b)^	26(22.2%)	29 (30.4%)	0.233

^(a)^ mean ± std. dev; ^(b)^ observed frequency (percentage); chi-square statistical test (either asymptotic, Fisher’s exact test, or Monte-Carlo simulation with 10,000 samples); ^(c)^ Mann–Whitney statistical test.

**Table 3:** Mann–Whitney U Test Results.

Variable	U	Z	*p*	R (Effect Size)
SF-36-mental component	5474.500	−0.187	0.852	−0.013
SF-36-physical component	5163.000	−0.888	0.374	−0.061
SCL-90—Anxiety	4671.000	−2.011	0.044	−0.138

Note: The Mann–Whitney U test was used to compare groups of participants with and without associated pathologies. The effect size r was calculated using the formula r=|Z|/√N . Confidence intervals represent a bias-corrected 95% CI based on Fisher’s z transformation.

**Table 4:** Descriptive Statistics (Mean Ranks).

Variable	Group	*N*	Mean Rank	Sum of Ranks
SF-36-mental component	Without pathologies	117	105.79	12,377.50
	With pathologies	95	107.37	10,200.50
SF-36-physical component	Without pathologies	117	103.13	12,066.00
	With pathologies	95	110.65	10,512.00
SCL-90—Anxiety	Without pathologies	117	98.92	11,574.00
	With pathologies	95	115.83	11,004.00

Note: *N* is the number of participants in each group. Mean ranks indicate score trends in each group, and the Mann–Whitney U test evaluates the differences between them.

**Table 5:** Spearman Correlations Between Physical and Mental Health and Anxiety Scores.

Variable	*N*	1	2	3
1. SF_mental	212	-	−0.126	0.083
2. SF_physical	212	−0.126	-	−0.164 *
3. SCL_anxiety	212	0.083	−0.164 *	-

Note. Correlations are Spearman’s *r_s_* coefficients. * *p* < 0.05. SF_mental = the Mental Component Summary of SF-36. SF_physical = the Physical Component Summary of SF-36. SCL_anxiety = the anxiety subscale of SCL-90-R.

**Table 6:** Spearman Correlations Between Physical and Mental Health and Anxiety Scores in the pathological group.

Variable	*N*	1	2	4
1. SF_mental	95	-	−0.258 *	0.086
2. SF_physical	95	−0.258 *	-	−0.267 **
4. SCL_anxiety	95	0.086	−0.267 **	-

Note. Correlations are Spearman’s *r_s_* coefficients. * *p* < 0.05, ** *p* < 0.01. SF_mental = the Mental Component Summary of SF-36. SF_physical = the Physical Component Summary of SF-36. SCL_anxiety = the anxiety subscale of SCL-90-R.

## Data Availability

The original contributions presented in this study are included in the article. Further inquiries can be directed to the corresponding author.
